# Genetic Downregulation of GABA_B_ Receptors from Oligodendrocyte Precursor Cells Protects Against Demyelination in the Mouse Spinal Cord

**DOI:** 10.3390/cells13232014

**Published:** 2024-12-05

**Authors:** Davide Gobbo, Phillip Rieder, Li-Pao Fang, Emeline Buttigieg, Moritz Schablowski, Elisa Damo, Nathalie Bosche, Eleonora Dallorto, Pascal May, Xianshu Bai, Frank Kirchhoff, Anja Scheller

**Affiliations:** 1 Department of Molecular Physiology, Center for Integrative Physiology and Molecular Medicine (CIPMM), Saarland University, 66421 Homburg, Germany; davide.gobbo@uks.eu (D.G.);; 2 Institut des Neurosciences de la Timone (INT), Aix-Marseille Université, CNRS UMR7289, 13005 Marseille, France; 3 Center for Gender-Specific Biology and Medicine (CGBM), Saarland University, 66421 Homburg, Germany; 4 Chengdu Center for Gender-Specific Biology and Medicine (CGBM Chengdu), Chengdu University of Traditional Chinese Medicine, Chengdu 610075, China; 5 Experimental Research Center for Normal and Pathological Aging, University of Medicine and Pharmacy of Craiova, 200349 Craiova, Romania

**Keywords:** spinal cord, GABA_B_ receptors, oligodendrocyte precursor cells, demyelination, cuprizone

## Abstract

GABAergic signaling and GABA_B_ receptors play crucial roles in regulating the physiology of oligodendrocyte-lineage cells, including their proliferation, differentiation, and myelination. Therefore, they are promising targets for studying how spinal oligodendrocyte precursor cells (OPCs) respond to injuries and neurodegenerative diseases like multiple sclerosis. Taking advantage of the temporally controlled and cell-specific genetic downregulation of GABA_B_ receptors from OPCs, our investigation addresses their specific influence on OPC behavior in the gray and white matter of the mouse spinal cord. Our results show that, while GABA_B_ receptors do not significantly alter spinal cord myelination under physiological conditions, they distinctly regulate the OPC differentiation and Ca^2+^ signaling. In addition, we investigate the impact of OPC-GABA_B_ receptors in two models of toxic demyelination, namely, the cuprizone and the lysolecithin models. The genetic downregulation of OPC-GABA_B_ receptors protects against demyelination and oligodendrocyte loss. Additionally, we observe the enhanced resilience to cuprizone-induced pathological alterations in OPC Ca^2+^ signaling. Our results provide valuable insights into the potential therapeutic implications of manipulating GABA_B_ receptors in spinal cord OPCs and deepen our understanding of the interplay between GABAergic signaling and spinal cord OPCs, providing a basis for future research.

## 1. Introduction

In the central nervous system (CNS), oligodendrocyte precursor cells (OPCs) represent a reservoir of new mature oligodendrocytes and exist in a 1:4 ratio with myelinating oligodendrocytes [1,2,3,4,5]. OPCs rapidly proliferate in response to injury [6], such as acute spinal cord injury [7], or in the context of neurodegenerative diseases affecting spinal cord myelination including chronic inflammatory diseases such as multiple sclerosis (MS) [8,9]. The loss of myelin occurring during MS is associated with immune-driven oligodendrocytes death [10,11,12] and results in axon exposure and the subsequent neurodegeneration [13,14]. As a response, OPCs migrate to the white matter lesions driven by resident micro- and astroglia, proliferate and differentiate [15,16,17,18], and participate in the spontaneous regeneration of myelin [19,20].

In line with the ability of OPCs to form synapses with neurons, both glutamate and GABA [21,22] have been identified as regulators of the oligodendrocyte-lineage cell proliferation [23,24,25,26], differentiation [23,24,26], and maturation [25] as well as myelination [24,25,27,28]. In particular, recent studies focusing on interneuron myelination and interneuron-OPC synapses in the brain have shed light on the importance of GABA signaling in the oligodendrocyte lineage [28,29]. The activation of OPC GABA_B_ receptors (GABA_B_Rs) increases OPC proliferation and migration in vitro [26,30]. Moreover, the GABA_B_R agonist baclofen enhances remyelination in the lysolecithin model of spinal cord demyelination in vivo [31]. Conversely, in the rat brain during development, GABA_B_R antagonism has been shown to increase OPC proliferation and decrease OPC maturation and myelination [32], hinting at a putative differential role of GABA_B_Rs on the physiology of oligodendrocyte-lineage cells based on the considered organism, region, or developmental stage. In OPCs, the activation of GABA_B_Rs results in the G_i/o_-protein-induced reduction in the intracellular levels of cyclic AMP [30], but an alternative G_q_ protein pathway linked to phospholipase C and intracellular Ca^2+^ has been suggested as well [22]. Indeed, OPCs respond to GABAergic neurons via intracellular Ca^2+^ oscillations [33,34]. Moreover, Ca^2+^ signaling has been linked to the regulation of myelination in oligodendrocyte-lineage cells [35,36,37,38] as well of the OPC fate [39,40].

The involvement of GABA_B_Rs as well as OPC Ca^2+^ signaling in the regulation of oligodendrocyte-lineage cell proliferation and fate points at their contribution to the pathological outcome of demyelination insults. Indeed, both pre- and postsynaptic GABAergic neurotransmission [41,42] as well as GABA levels [43,44] are altered in the brains of MS patients and the GABA_B_R agonist baclofen itself is currently used as a therapeutical agent for spasticity in MS [45]. The specific role of GABA_B_Rs in OPCs within spinal cord physiology and pathology remains poorly understood in vivo. In this study, we address this gap by employing the genetic downregulation of GABA_B_Rs from OPCs, aiming to elucidate their contribution to cell proliferation, differentiation, myelination, and OPC Ca^2+^ signaling. Additionally, taking advantage of the cuprizone and lysolecithin models of toxic demyelination, we explore the role of OPC GABA_B_Rs in the cellular response to pathological demyelinating insults of the spinal cord white matter.

## 2. Materials and Methods

### 2.1. Animals

Mice were maintained in the animal facility of the Center for Integrative Physiology and Molecular Medicine (CIPMM, University of Saarland, Homburg). Humidity and temperature were maintained at 45–65% and 20–24 °C and the facility was kept under a 12 h light–dark cycle. All mice received food ad libitum (standard autoclaved rodent diet, Ssniff Spezialdiäten, Soest, Germany) and autoclaved tap water. Knock-in NG2-CreERT2 mice (Cspg4^tm1.1(cre/ERT2)Fki^, MGI: 5566862) [4] were crossbred to Rosa26 reporter mice (Gt(ROSA)26Sor^tm1(CAG-GCaMP3)Dbe^, MGI: 5659933) [46] and Gabbr1^tm2Bet^ mice (MGI: 3512742) [47] and maintained in a C57BL/6N background. To induce genetic recombination, tamoxifen (Carbolution, Neunkirchen, Germany) solved in Mygliol (Caesar&Loretz GmbH, Hilden, Germany) was administered intraperitoneally (i.p.) for five consecutive days (once per day, 100 mg/kg body weight) [48] at 10 weeks of age. Both control and conditional knock-down mice were heterozygous for NG2-CreERT2 (*ct2/+*) and homozygous floxed for GCaMP3 (*fl/fl*), and were wild-type (*+/+*) or floxed (*fl/fl*) for *gabbr1*, respectively. In Supplementary Figure S2, early tamoxifen administration was performed at one week of age (two consecutive days, once per day, 100 mg/kg body weight) or at four weeks of age (five consecutive days, once per day, 100 mg/kg body weight). Two-photon laser-scanning microscopy (2P-LSM) and immunohistochemistry (IHC) were performed at least two weeks after beginning of the tamoxifen treatment (control and cKD mice). For the demyelination study, 12-week-old male and female mice were fed with standard powder food containing 0.3% cuprizone for one week and 0.2% cuprizone for other two weeks (Bis(cyclohexanone)oxaldihydrazone, Carbolution Chemicals, St. Ingbert, Germany). The diet was freshly prepared and changed every day. After three weeks of diet, the animals were fed standard food until further analysis. This protocol has been previously established and was used to address demyelination in the brain resulting in a 90% loss of oligodendrocytes in the middle part of the corpus callosum [49]. 2P-LSM and IHC analysis for cuprizone treated-mice and age matched controls were performed on 15–16-week-old mice. 

The Number of animals used for each experiment is listed in the corresponding figure legend. In this study, animals of both sexes were used and were equally represented. No sex difference was observed across the study and data were accordingly not segregated into two groups.

### 2.2. Laminectomy and Spinal Window Implantation

All surgical sections were realized in animals under inhalational anesthesia (1.5–2% isoflurane, 66% O_2_, and 33% N_2_O) and the animal’s eyes were covered by Bepanthen (Bayer, Leverkusen, Germany). Surgeries were executed as previously described [50] to gain access to T12–L2 vertebrae, and, by laminectomy approach, L4–S1 spinal segments could be exposed. A modified coverslip was fit on the spinal cord and animals were treated with analgesic and antiphlogistic agents for three consecutive days [51].

### 2.3. Acute Lysolecithin Incubation

The procedure for acute incubation with lysolecithin (LPC, 1% *v/v* in NaCl 0.9%, Merck KGaA, Darmstadt, Germany) was performed as previously described [52]. After laminectomy, the exposed dura mater was opened with a fine needle, washed with artificial cerebrospinal fluid (aCSF), and incubated for one hour with 20 μL LPC. Afterwards, the LPC was dried out and the tissue was thoughtfully washed with aCSF. The window was placed and sealed as previously described. Animals were perfused one week later.

### 2.4. Two-Photon Laser-Scanning Microscopy

In vivo recordings were performed using a custom-made two-photon laser-scanning microscope (2P-LSM), equipped with a mode-locked Ti:sapphire femtosecond pulsed laser, Vision II (Coherent, St. Clara, CA, USA) [53], in combination with ScanImage software (v5.7) [54], as previously described [50]. For transgenic GCaMP3 excitation, the laser wavelength was set to 890 nm and the power was adjusted from 8 to 60 mW, depending on the imaging depth in the tissue. 2P-LSM was performed on the white matter of the dorsal funiculus up to a 100–150 µm depth by using a long-distance W Plan-Apochromat 20× 1.0 NA DIC objective (Zeiss, Oberkochen, Germany). Areas of white matter were recorded as uniformly spaced planes of field of views with 256 × 256 pixel per image, and 1.4 µs pixel dwell time, and GCaMP3 emission was acquired using a 500/24 nm band pass filter, detected by a photomultiplier tube H10770PB-40 (Hamamatsu Photonics, Hamamatsu, Japan). During 2P-LSM in vivo recordings, animals were kept under inhalation anesthesia with 1.5% isoflurane on a heating plate.

### 2.5. Automated ROA-Based Detection and Analysis of Ca^2+^ Events

Ca^2+^-event analysis was performed using the custom-made MATLAB-based analysis software MSparkles (https://gitlab.com/Gebhard/MSparkles, accessed on 26 November 2024) [55] as previously described [50]. Shortly, fluorescence fluctuations at basal Ca^2+^ concentrations (F0) were computed along the temporal axes of each individual pixel, by fitting a polynomial of user-defined degree in a least-squares sense. The range projection of ΔF/F0 was then used to identify local fluorescence maxima, serving as seed points for simultaneous, correlation-based region growing (minimum ROA area, 5 µm^2^; temporal correlation threshold, 0.2). Prior to F0 estimation, image stacks were denoised using the PURE-LET algorithm [56] as well as a temporal median filter to correct small motion artefacts and simultaneously retain sharp transient edges. Based on the pre-processed data (F), Ca^2+^ event detection and analysis were performed on the normalized dataset (ΔF/F0).

### 2.6. Immunohistochemistry

Anesthetized animals were transcardially perfused with phosphate-buffered saline (PBS) and tissue was fixed by 4% formaldehyde perfusion. After 24 h post fixation in 4% formaldehyde, T13–L1 spinal cord segments were dissected and detached from meninges. The spinal cord tissue was maintained in PBS and cut in transversal sections (40 µm) using a vibratome (VT1000 S) (Leica, Nußloch, Germany). Free-floating slices were processed as described before [57]. Briefly, incubation in blocking solution (Triton X-100, horse serum and PBS) at room temperature (RT) was followed by primary antibody solution incubation overnight at 4 °C for detection of the following markers: monoclonal mouse: anti-APC CC-1 (1:50, Cat. No. OP80, Merck, KGaA, Darmstadt, Germany; anti-MBP (1:500, clone SMI99, Biolegend, San Diego, CA, USA); monoclonal rat: anti-Ki67 (1:500, Cat. No. 14-5698-82, Thermo Fisher Scientific, Waltham, MS, USA); polyclonal rabbit: anti-MyRF (1:500, Cat. No. OB-PRB007, Oasis Biofarm, Hangzhou, China), and anti-Olig2 (1:1000, Cat. No. AB9610, Merck, KGaA, Darmstadt, Germany); polyclonal goat: anti-GFAP (1:1000, ab53554, Abcam, Cambridge, UK), anti-Iba1 (1:1000, ab5076, Abcam, Cambridge, UK), and anti-PDGFRα (1:1000, Cat. No. AF1062, R&D Systems, Minneapolis, MN, USA); and polyclonal chicken: anti-GFP (1:1000, Cat. No. 10524234, Fisher Scientific GmbH, Schwerte, Germany), and anti-NF (1:500, ab4680, Abcam, Abcam, Cambridge, UK). For MBP myelin staining, sections were pretreated with ethanol (>99%) for 10 min prior to incubation in blocking solution. Secondary antibodies (donkey secondary antibodies conjugated with Alexa488, Alexa555, Alexa647, and Alexa750; 1:2.000 in PBS; Invitrogen, Grand Island, NY, USA and goat secondary antibody conjugated with Cy5, 1:1000 in PBS, Biozol, Eching, Germany) were incubated for 2 h at RT in the dark.

### 2.7. Epifluorescence Analysis and Cell Counting

Detection was executed with the fully automated epifluorescence slide scanner microscope AxioScan.Z1 (Zeiss, Oberkochen, Germany) equipped with a Colibri 7 LED system, a Plan-Apochromat 20×/0.8 objective, and appropriate beam splitters for DAPI (405 nm), Alexa488 (495 nm), Alexa555 (573 nm), Alexa647/Cy5 (652 nm), and Alexa750 (762 nm). Image stacks (5 µm, variance projection) were recorded and analyzed with ZEN 3.1 blue edition (Zeiss, Oberkochen, Germany). For cell counting, OPCs were identified using PDGFRα, oligodendrocytes using APC-CC1 and MyRF and cells of the oligodendrocyte lineage using Olig2. Recombined cells were identified by the co-expression of GCaMP3 (GFP^+^ cells) to differentiate them from non-recombined (GFP^−^) cells used as internal controls.

### 2.8. Confocal Analysis

Confocal magnification images (512 × 512 pixel, 1.54 µs pixel dwell time) were acquired with the confocal laser-scanning microscope cLSM 880 (Zeiss, Oberkochen, Germany) using a Plan-Apochromat 63×/1.4 Oil DIC M27 objective, argon (488 nm) and helium–neon laser (542 nm) excitation, and appropriate beam splitters. The setup and image acquisition were controlled by ZEN black 2.3 software (Zeiss, Oberkochen, Germany). For the evaluation of the relative axonal MBP area, the area delimited by the MBP signal surrounding single axons was divided by the neurofilament-positive area enclosed within the MBP signal.

### 2.9. Magnetic Cell Separation (MACs)

Magnetic cell separation (MACs) of OPCs was performed as previously described [28]. Briefly, anesthetized animals were perfused using ice-cold Hank’s balanced salt solution without Ca^2+^ and Mg^2+^ (HBSS, Life Technologies, Carlsbad, CA, USA) and the spinal cord was dissected on ice. The sorting was performed on the basis of the manufacturer’s instructions (Miltenyi Biotec, Bergisch-Gladbach, Germany) with minor adjustments: First, debris removal (130-107-677) and resuspension in re-expressing medium (NeuroBrew-21, 1:50, Cat. No. 130-093-566 and 200 mM L-glutamine, 1:100, Merck KGaA, Darmstadt, Germany; in MACs Neuro Medium, Cat. No. 130-093-570) at 37 °C for 30 min were performed. Subsequently, myelin removal (130-096-731) for 15 min at 4 °C and incubation in Fc-receptor blocker for 10 min at 4 °C were performed. Finally, cells were incubated at 4 °C with 10 µL microbead mixture with antibodies against CD140 (130-101-502), NG2 (130-097-170), and O4 (130-096-670) in a 1:1:1 ratio. MAC-sorted OPCs and flow samples were lysed in RIPA buffer (Thermo Fisher Scientific, Waltham, MS, USA) and stored at −80 °C until further analysis.

### 2.10. Real-Time PCR

For quantitative real-time PCR (qRT-PCR), mRNA was extracted using the NucleoSpin RNA Plus XS kit (Macherey-Nagel, Düren, Germany) and cDNA was produced using the Omniscript kit (QIAGEN, Venlo, The Netherlands). qRT-PCR was performed using the EvaGreen kit (Axon Labortechnik, Kaiserslautern, Germany) with a CFX96 Real Time System (BioRad, Feldkirchen, Germany). The primer sequences used are listed in Supplementary Table S1.

### 2.11. Software

For 2P-LSM acquisition, the open-source MATLAB-based software application ScanImage v5.7^®^ (Vidrio Technologies, Ashburn, VA, USA) [54] was used. The custom-made MATLAB-based software MSparkles (https://gitlab.com/Gebhard/MSparkles, accessed on 26 November 2024), GraphPad Prism 9, and Microsoft Office Excel 2019 were used for data analysis. Immunohistochemical data were collected, visualized, and modified using the ZEN imaging software blue and black editions (3.1 + 2.3) (Zeiss, Oberkochen, Germany) and the ImageJ collection Fiji (https://imagej.net/ij/, accessed on 26 November 2024). For figure layout, the Adobe Creative Suite 2021–2024 was used (Adobe InDesign^®^, Adobe Illustrator^®^, and Adobe Photoshop^®^). Administration of the animals was performed with PyRAT (Version 4.6.1-859), a Python-based Relational Animal Tracking software (Scionics Computer Innovation; Dresden, Germany).

### 2.12. Statistics

Unless otherwise stated, data were analyzed using a Shapiro–Wilk normality test and, accordingly, represented as mean ± SEM or median with interquartile range of single mice. Single datasets belonging to the same animal were similarly analyzed and represented as mean or median. Data were compared using appropriate parametric or non-parametric tests in line with the result of the Shapiro–Wilk normality test. Test details are given in the corresponding figure legend. The following *p*-values were used for statistical analysis: *, # *p* < 0.05; ** *p* < 0.01; *** *p* < 0.001, and **** *p* < 0.0001.

## 3. Results

### 3.1. GABA_B_R Downregulation in OPCs Affects OPC Differentiation in the Spinal Cord Without Compromising Myelination

**Figure 1:**
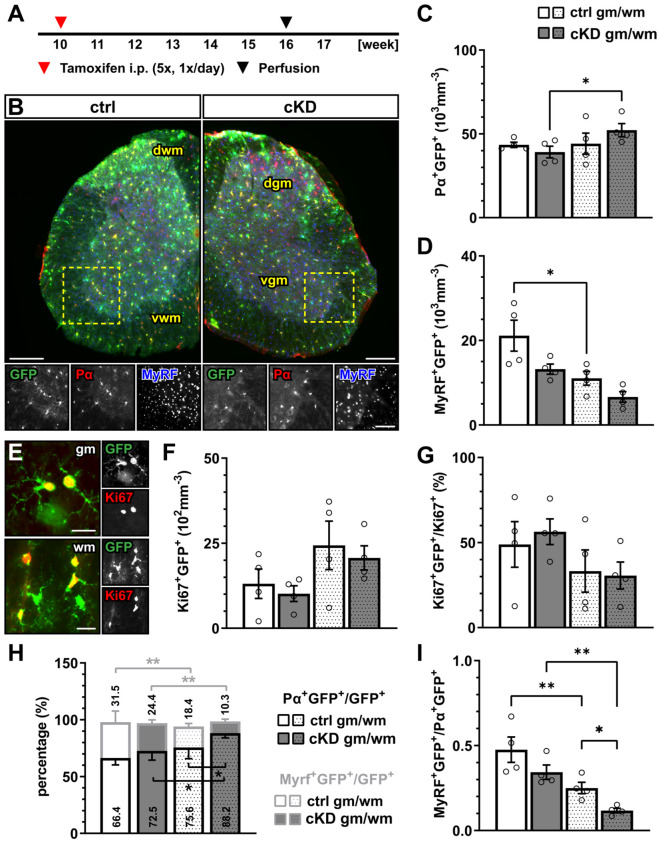
GABA_B_R downregulation in OPCs does not affect oligodendrocyte-lineage cell proliferation but differentiation. (**A**) Experimental design for tamoxifen-induced GABA_B_R downregulation and GCaMP3 (GFP) expression in NG2^+^ OPCs, perfusion, and immunohistochemical analysis (IHC). (**B**) IHC of spinal cord tissue from control (ctrl) and conditional knock-down (cKD) mice stained for GFP (green), PDGFRα (Pα, red), and MyRF (blue). dwm, dorsal white matter; dgm, dorsal gray matter; vgm, ventral gray matter; vwm, ventral white matter. Scale bar, 200 µm. Recombined OPC (Pα^+^GFP^+^, (**C**)) and mature oligodendrocyte (MyRF^+^GFP^+^, (**D**)) cell density. (**E**) IHC analysis of proliferating cells (Ki67^+^, red). Scale bar, 20 µm. Recombined proliferating cell density (**F**) and proportion of recombined cells (Ki67^+^GFP^+^) in the total pool of proliferating cells (Ki67^+^) (**G**). (**H**) Proportion of recombined OPCs and mature oligodendrocytes in the total pool of recombined cells. (**I**) Ratio between recombined oligodendrocytes and recombined OPCs. Data are represented as mean ± SEM and are derived from N = 3–4 mice (*n* = 12 FOVs). Data were analyzed using a two-way ANOVA with multiple-comparisons test. * *p* < 0.05; ** *p* < 0.01.

To investigate the contribution of metabotropic GABA_B_ receptors (GABA_B_R) in oligodendrocyte-precursor cells (OPCs), we induced the time-controlled and cell-specific GABA_B_R downregulation in NG2^+^ OPCs coupled to the expression of the genetically encoded lineage tracer and Ca^2+^ indicator GCaMP3 (GABA_B_R cKD mice). To this aim, 10-week-old mice were injected i.p. for five consecutive days with tamoxifen at a daily dose of 100 mg/kg body weight (Figure 1A), leading to the reduction in the gabbr1 mRNA levels in sorted OPCs by ~ 40% (*p* = 0.034) (Supplementary Figure S1A–D) and the recombination of more than 80% of PDGFRα^+^ OPCs two weeks after the first TAM injection (Supplementary Figure S1E,F). Although a longer latency between the knock-down induction and analysis results in a higher efficiency [48], an earlier tamoxifen administration also resulted in an increased proportion of recombined mature oligodendrocytes as a result of the differentiation of the recombined OPCs (Supplementary Figure S2), whereas our chosen experimental design reduced the proportion of the recombined MyRF^+^ mature oligodendrocyte recombination to ~5–6% in the gray matter and to ~2–3% in the white matter (Supplementary Figure S1G).

In the gray and white matter of lumbar spinal cord tissue, the genetic downregulation of GABA_B_R from OPCs did not affect the density of recombined PDGFRα^+^ OPCs (Figure 1A–C) or the density of the recombined MyRF^+^ mature oligodendrocytes (Figure 1D) between the control (ctrl) and GABA_B_R-conditional knock-down mice (cKD). Similarly, non-recombined cells did not differ between the two groups (Supplementary Figure S3). In line with these results, no overall alteration of the proliferation rate was detected using the proliferation nuclear marker Ki67 (Figure 1E,F). Finally, to evaluate the differentiation of the recombined OPCs, we quantified the percentage of the recombined OPCs (PDGFRα^+^GFP^+^) and recombined oligodendrocytes (MyRF^+^GFP^+^) on the total number of recombined cells (GFP^+^). Specifically, in the white matter, the relative number of PDGFRα^+^GFP^+^ was increased in cKD mice (ctrl, 75.62 ± 4.95%; cKD, 88.20 ± 2.08%; *p* = 0.011), suggesting a reduced OPC differentiation in cKD mice (Figure 1H). On the other hand, the relative number of MyRF^+^GFP^+^ did not differ between ctrl and cKD mice. The increased PDGFRα^+^GFP^+^ relative number in the white matter of cKD mice was reflected by a reduced MyRF^+^GFP^+^/PDGFRα^+^GFP^+^ ratio representing the number of mature oligodendrocytes produced by a single recombined OPC (Figure 1I).

**Figure 2:**
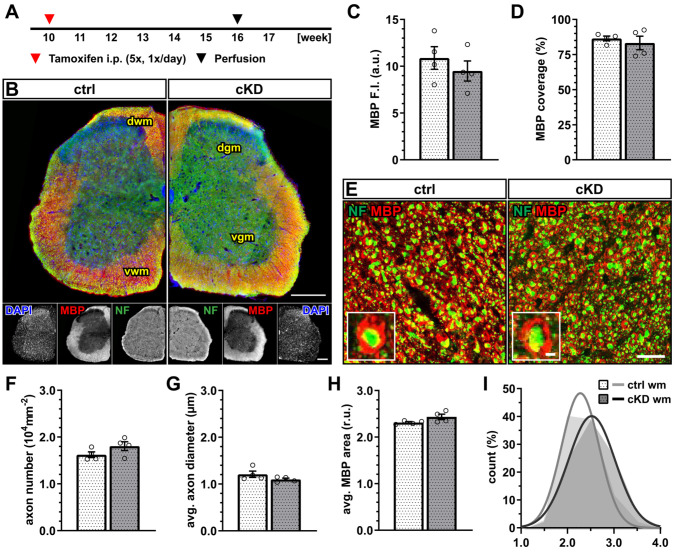
No changes in spinal white matter myelination after GABA_B_R downregulation. (**A**) Experimental design for tamoxifen-induced GABA_B_R downregulation and GCaMP3 (GFP) expression in NG2^+^ OPCs, perfusion, and IHC. (**B**) IHC of spinal cord tissue from ctrl and cKD mice stained for DAPI (blue), MBP (red), and neurofilament (green). Scale bar, 200 µm. (**C**) Mean fluorescence intensity (F.I.) of white matter MBP signal and (**D**) MBP coverage expressed as the percentage of the MBP^+^ area in selected FOVs. (**E**) Confocal analysis of white matter spinal cord tissue stained for MBP (red) and neurofilament (green). Scale bar, 10 µm (magnification: 2 µm). (**F**) Axonal density and (**G**) average axonal diameter. (**H**) Average relative axonal MBP area and (**I**) associated histogram of distribution (as filled area) as well as non-linear Gaussian fit (as solid line). Data are represented as mean ± SEM and are derived from N = 4 mice (*n* = 16 FOVs). In (**H**,**I**), data are derived from *n* = 160 axons per group. Data were analyzed using an unpaired *t* test. In (**I**), non-linear fitting was performed using a least-squares fitting with no weighting method and compared using the extra-sum-of-squares F test.

Next, we investigated whether OPC-GABA_B_R cKD mice display any myelin alterations in the white matter of the spinal cord (Figure 2A,B). No difference was detected in terms of the mean fluorescence intensity of the myelin basic protein (MBP) (Figure 2C) as well as in terms of the myelinated area (Figure 2D). To exclude that these results mask underlying subtle differences in axonal myelination, we performed the higher-magnification confocal acquisition of MBP, coupled with the immunohistochemical detection of neurofilament-positive neuronal axons (Figure 2E). The results showed no difference in axon number (Figure 2F) as well as average axon diameter (Figure 2G). Accordingly, we calculated the per-axon relative MBP area (as the ratio of the area delimitated by the MBP^+^ signal normalized on the neurofilament-positive area) and its distribution and found no difference between ctrl and cKD mice (Figure 2H).

These data, therefore, suggest that the genetic downregulation of OPC-GABA_B_Rs is not associated with obvious alterations in the physiological level of cell proliferation and the differentiation of the oligodendrocyte lineage, as well as myelination within the spinal cord.

### 3.2. Oligodendrocyte Lineage Cells Respond Differently to Cuprizone Treatment upon OPC-GABA_B_R Downregulation

**Figure 3:**
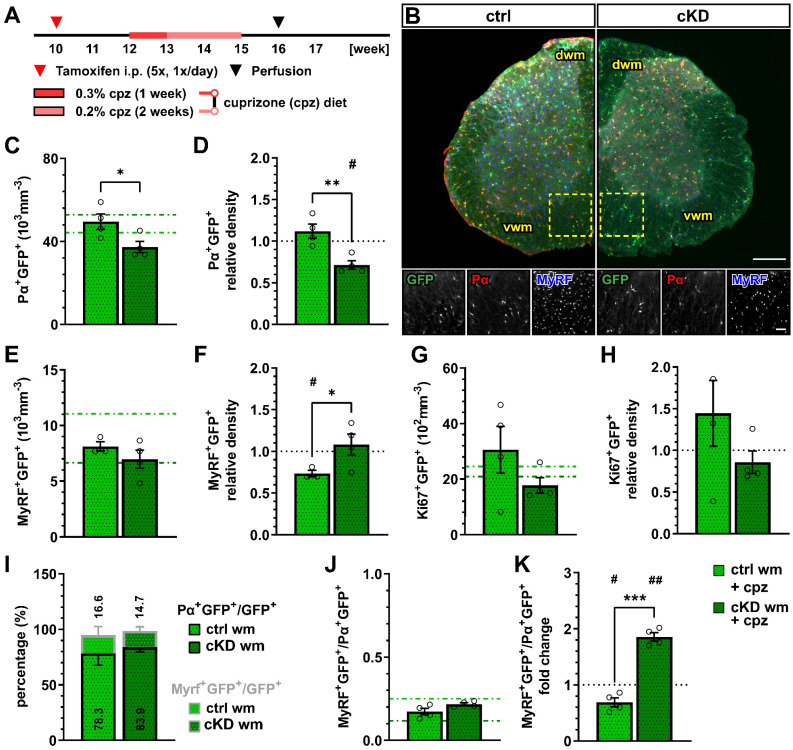
OPC-GABA_B_R downregulation leads to reduced OPC density and unaltered mature oligodendrocyte density after cuprizone treatment. (**A**) Experimental design for tamoxifen-induced GABA_B_R downregulation and GCaMP3 (GFP) expression in NG2^+^ OPCs, cuprizone (cpz) treatment, perfusion, and IHC analysis. (**B**) IHC from cuprizone-treated ctrl and cKD mice stained for GFP (green), PDGFRα (Pα, red), and MyRF (blue). Scale bar, 200 µm (overview), 100 µm (zoom-in). Recombined OPC (Pα^+^GFP^+^, (**C**)) cell density and fold-change (**D**) compared to untreated ctrl and cKD mice. Recombined mature oligodendrocyte (MyRF^+^GFP^+^, (**E**)) cell density and fold-change (**F**). Recombined proliferating (Ki67^+^GFP^+^, (**G**)) cell density and fold-change (**H**). (**I**) Proportion of recombined OPCs and mature oligodendrocytes in the total pool of recombined cells. (**J**) Ratio between recombined oligodendrocytes and recombined OPCs and (**K**) fold-change compared to untreated ctrl and cKD mice. Data are represented as mean ± SEM and are derived from N = 3–4 mice (*n* = 12 FOVs). In (**C**,**E**,**G**,**J**), data from untreated groups are overlapped as dotted lines (ctrl, light green; cKD, dark green). In (**D**,**F**,**H**,**K**), the black dotted line corresponding to the non-treated ctrl and cKD (fold change = 1) is included as reference. Data were analyzed using an unpaired *t* test (**C**–**I**,**K**) or a two-way ANOVA with multiple-comparisons test (**J**). For each group in (**D**,**F**,**H**,**K**), data were tested using a one-sample *t* test to compare their mean with the hypothetical value μ = 1. *, # *p* < 0.05; **, ## *p* < 0.01; *** *p* < 0.001.

The cuprizone model is a copper-chelating toxic model used to induce demyelination in the CNS, mimicking several aspects of human multiple sclerosis (MS), including demyelination, oligodendrocyte death, astrogliosis, microgliosis, OPC proliferation, and maturation [58,59]. The cuprizone-induced response in the spinal cord has been reported to be reduced compared to the other regions of the forebrain [60,61,62]. By virtue of the previous reports on the applicability of the model in the spinal cord, we modified the previously used 0.2% *w*/*w* cuprizone powder feeding model and fed the 12-week-old mice with a dose of 0.3% *w*/*w* cuprizone for one week and with a dose of 0.2% *w*/*w* for the two following weeks (Figure 3A).

One week after the withdrawal of the cuprizone diet, we performed an immunohistochemical analysis of the oligodendrocyte lineage (Figure 3B). The recombined OPC density in the white matter of cuprizone-treated OPC-GABA_B_R cKD mice (37.24 ± 2.73 x10^3^ mm^−3^) was reduced compared to cuprizone-treated controls (49.49 ± 3.71 x10^3^ mm^−3^, *p* = 0.031) (Figure 3C). Moreover, compared to the untreated control mice, the cuprizone-treated controls showed no change in the density of recombined OPCs, whereas cKD mice displayed a ~30% decrease in OPC cell density (0.71 ± 0.05, *p* = 0.012) (Figure 3D). Interestingly, the cell density of non-recombined OPCs was increased in cuprizone-treated OPC-GABA_B_R cKD mice compared to cuprizone-treated control mice (Supplementary Figure S4A–C), as well as untreated cKD mice (Supplementary Figure S4D,E), suggesting that non-recombined OPCs may proliferate to compensate for the reduced density of GABA_B_R-lacking OPCs.

No difference was observed in terms of the recombined mature oligodendrocyte cell density between the cuprizone-treated control (8.12 ± 0.43 × 10^3^ mm^−3^) and cKD mice (6.97 ± 0.82 × 10^3^ mm^−3^, *p* = 0.628) (Figure 3E). To note, however, only control mice showed a reduction in MyRF^+^GFP^+^ cell density upon cuprizone treatment (0.73 ± 0.04, *p* = 0.021), whereas OPC-GABA_B_R cKD mice did not show any difference compared to untreated animals (1.05 ± 0.12, *p* = 0.723) (Figure 3F). These data suggest that the downregulation of GABA_B_Rs from the oligodendrocyte-lineage cells may be associated with enhanced OPC differentiation upon cuprizone treatment, resulting in an increased mature oligodendrocyte density. Interestingly, the density of non-recombined mature oligodendrocytes in cKD mice was reduced by the cuprizone treatment similarly to control mice (Supplementary Figure S4D,E), indicating that the protective effect of the genetic downregulation of OPC-GABA_B_Rs is restricted to recombined cells and their progeny.

With respect to cell proliferation, the density of recombined Ki67^+^ cells, indicative of proliferating recombined OPCs, did not differ either between the cuprizone-treated control and cKD mice (Figure 3G) or between the treated and untreated groups (Figure 3H). To note, no difference was detected in the relative number of PDGFRα^+^GFP^+^ or MyRF^+^GFP^+^ cells (Figure 3I) and the MyRF^+^GFP^+^/PDGFRα^+^GFP^+^ ratio between controls and cKD mice (Figure 3J). On the other hand, the MyRF^+^GFP^+^/PDGFRα^+^GFP^+^ ratio was almost doubled in cKD mice compared to untreated mice (1.86 ± 0.07, *p* = 0.001), whereas it was reduced in control mice (0.69 ± 0.08, *p* = 0.029) (Figure 3K), in line with the enhanced OPC differentiation after the loss of the OPC-GABA_B_R.

The astro- and microgliosis, in response to cuprizone treatment, were evaluated by means of an immunohistochemical analysis of the glial fibrillary acidic protein (GFAP) and the ionized calcium-binding adapter molecule 1 (Iba1), respectively (Supplementary Figure S5A,B). In line with the reduced effect of cuprizone on spinal cord tissue published before, no clear white matter astroglial activation or difference between control and cKD mice were detected upon cuprizone treatment (Supplementary Figure S5C,D). The same was true for the number of Iba1^+^ cells (Supplementary Figure S5E), which did not increase upon cuprizone treatment, nor was different between the groups (Supplementary Figure S5F), further highlighting the subtle effect of CPZ in the spinal cord.

Given the enhanced number of oligodendrocytes and the increased OPC differentiation upon cuprizone treatment, these results suggest that the downregulation of GABA_B_Rs from OPCs protects against demyelination in the spinal cord.

### 3.3. Cuprizone-Induced Myelin Loss in the Spinal Cord Is Ameliorated in OPC-GABA_B_Rs cKD Mice

**Figure 4:**
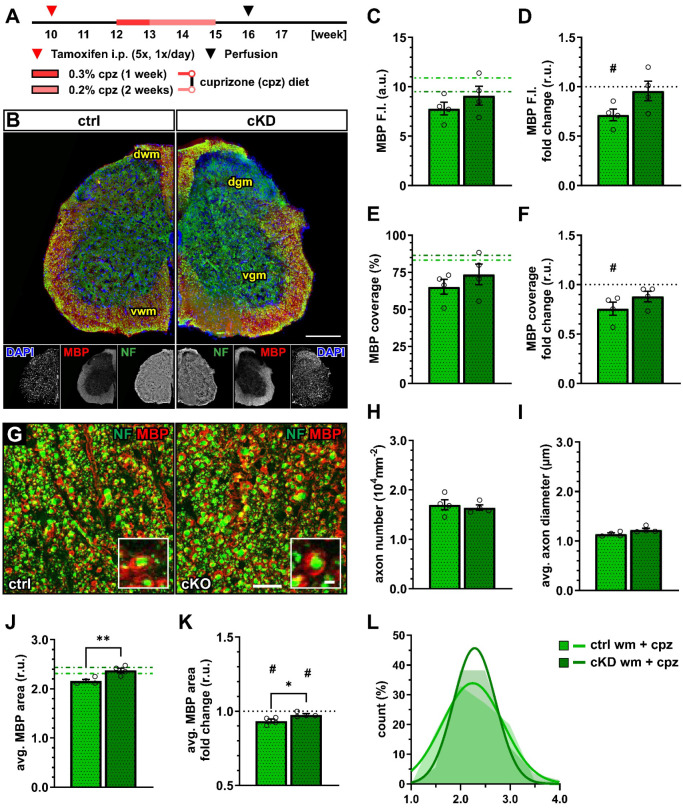
OPC-GABA_B_R cKD protects against cuprizone-induced myelin loss in the spinal cord. (**A**) Experimental design for tamoxifen-induced GABA_B_R downregulation and GCaMP3 (GFP) expression in NG2^+^ OPCs, cuprizone (cpz) treatment, perfusion, and IHC. (**B**) IHC from ctrl and cKD mice stained for DAPI (blue), MBP (red), and neurofilament (green). Scale bar, 200 µm. (**C**) Mean fluorescence intensity (F.I.) of white matter MBP signal and (**D**) fold-change compared to untreated ctrl and cKD mice. (**E**) MBP coverage expressed as the percentage of the MBP^+^ area in selected FOVs and (**F**) fold-change compared to untreated ctrl and cKD mice. (**G**) Confocal analysis of white matter spinal cord tissue stained for MBP (red) and neurofilament (green). Scale bar, 10 µm (magnification: 2 µm). (**H**) Axonal density and (**I**) average axonal diameter. (**J**) Average relative axonal MBP area, (**I**) fold-change, and (**L**) associated histogram of distribution (as filled area) as well as non-linear Gaussian fit (as solid line). Data are represented as mean ± SEM and are derived from N = 4 mice (*n* = 16 FOVs). In (**J**,**K**), data are derived from *n* = 160 axons per group. In (**C**,**E**,**J**), data from untreated groups are overlapped as dotted lines (ctrl, light green; cKD, dark green). In (**D**,**F**,**K**), the black dotted line corresponding to the non-treated ctrl and cKD (fold change = 1) is included as reference. Data were analyzed using an unpaired *t* test. For each group in (**D**,**F**,**K**), data were tested using a one-sample *t* test to compare their mean with the hypothetical value μ = 1. In (**L**), non-linear fitting was performed using a least-squares fitting with no weighting method and compared using the extra-sum-of-squares F test. *, # *p* < 0.05; ** *p* < 0.01.

Next, we evaluated the effect of the cuprizone on myelin in control and OPC-GABA_B_R cKD mice (Figure 4A,B). Although the mean MBP fluorescence intensity was only slightly enhanced in cKD compared to control mice (ctrl, 7.80 ± 0.65; cKD, 9.10 ± 0.94; *p* = 0.371) (Figure 4C), MBP was reduced compared to untreated conditions specifically in control mice (0.72 ± 0.06, *p* = 0.018), but unaltered in cKD mice (0.96 ± 0.10, *p* = 0.703) (Figure 4D). Similar considerations hold true for the MBP coverage (ctrl, 65.19 ± 4.99; cKD, 73.56 ± 7.03; *p* = 0.260) (Figure 4E), which was reduced in control mice (0.76 ± 0.07, *p* = 0.036) and left unaltered in cKD mice (0.88 ± 0.05, *p* = 0.107) (Figure 4F).

A confocal analysis of myelinated axons (Figure 4G) showed no difference in terms of axon density (Figure 4H) as well as average axonal diameter (Figure 4I), indicating no difference in axonal survival or structure between control and cKD mice and their response to the toxicity of cuprizone. On the other hand, the average relative MBP area in cKD mice (2.37 ± 0.05) was higher than under control conditions (2.16 ± 0.03; *p* = 0.002) indicating higher myelination in the cKD mice after cuprizone treatment (Figure 4J), in line with the higher MBP fluorescence. Compared to untreated conditions, both groups displayed a reduced average relative MBP area (ctrl: 0.93 ± 0.01, *p* = 0.016; cKD: 0.98 ± 0.01, *p* = 0.050) but the reduction in cKD mice was lower than control mice (*p* = 0.033) (Figure 4K,L). These data are, therefore, in line with a protective role of OPC GABA_B_R downregulation to the pathological alteration of myelin quality associated with the cuprizone treatment.

### 3.4. Acute Myelin Loss upon Lysolecithin Incubation Is Reduced in OPC-GABA_B_R cKD Mice

**Figure 5:**
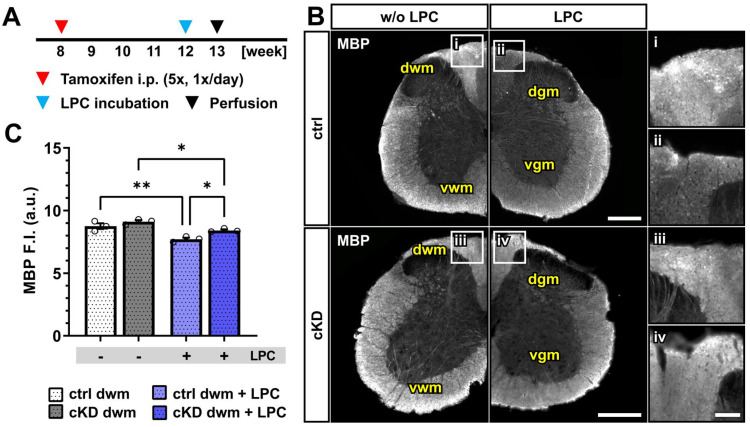
Acute myelin loss upon lysolecithin incubation reduced in OPC-GABA_B_R cKD mice. (**A**) Experimental design for tamoxifen-induced GABA_B_R downregulation and GCaMP3 (GFP) expression in NG2^+^ OPCs, lysolecithin (LPC) incubation, perfusion, and IHC. (**B**) IHC of spinal cord tissue from ctrl and cKD mice stained for MBP and magnified view of dwm incubated with saline (**i**,**iii**) or LPC (**ii**,**iv**). Scale bar, 200 µm (magnification: 50 µm). (**C**) Mean fluorescence intensity (F.I.) of white matter MBP signal. Data are represented as mean ± SEM and are derived from N = 3 mice (*n* = 12 FOVs). Data were analyzed using a two-way ANOVA with multiple-comparisons test. * *p* < 0.05; ** *p* < 0.01.

To confirm the protective effect of OPC-GABA_B_R loss on demyelinating insults, we took advantage of an acute model of demyelination, namely, the incubation with the gliotoxic membrane-dissolving compound lysolecithin (or lysophosphatidylcholine, LPC) [52,63]. The focal timely-controlled application of LPC on exposed FV

s of the lumbar spinal cord was used to evaluate the effect of acute demyelinating insults on the spinal dorsal white matter in control and cKD mice (Figure 5A). Contrary to the cuprizone model, LPC directly acts on immune cells, being a direct activator for monocytes, macrophages, and lymphocytes [64,65,66]. Therefore, inflammation is a predominant component of the model next to the direct detergent effect of LPC on oligodendrocytes. In line with this, we detected a strong glial response upon LPC incubation on the superficial layers of the dorsal white matter tracts in control and cKD mice (Supplementary Figure S6A,B), both in terms of astroglial GFAP expression (Supplementary Figure S6C,D) and Iba1^+^ microglia activation (Supplementary Figure S6E,F).

Upon LPC incubation, control mice showed a small but significant reduction in the mean MBP fluorescence intensity (~12%) restricted to the dorsal white matter (w/o LPC, 8.75 ± 0.23; LPC, 7.71 ± 0.13; *p* = 0.001). OPC-GABA_B_R cKD mice were also associated with a small MBP reduction (~8%; w/o LPC, 9.11 ± 0.12; LPC, 8.4 ± 0.08; *p* = 0.011) (Figure 5B,C) but to a lesser extent compared to LPC-treated control mice (*p* = 0.011). As was the case for the cuprizone model, the data are in line with a putative protective role of OPC-GABA_B_R-downregulation to demyelinating insults. Nevertheless, further assessment is required to address the specific role of OPC-GABA_B_Rs in the LPC model of demyelination.

### 3.5. Spinal Cord OPCs Display Altered Ca^2+^ Dynamics in OPC-GABA_B_R cKD Mice

**Figure 6:**
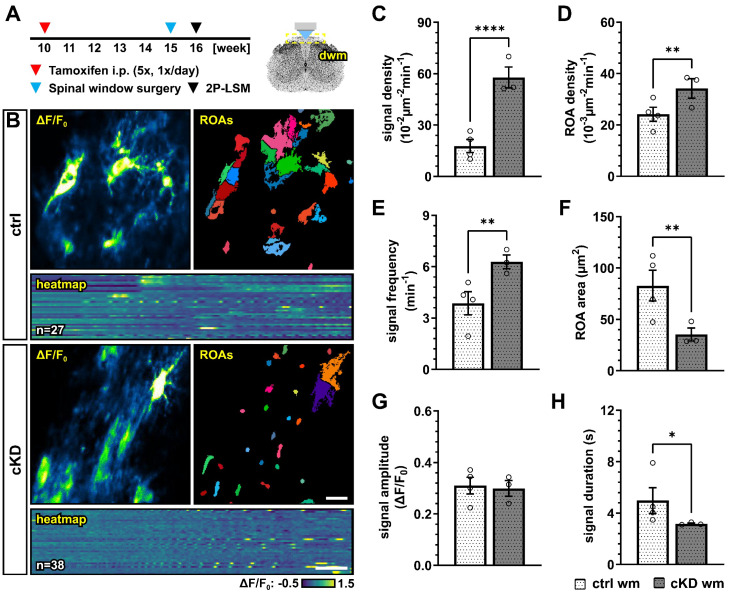
GABA_B_R cKD mice display aberrant Ca^2+^ dynamics in OPCs with increased signal frequency and density but reduced spatial and temporal profiles. (**A**) Experimental design for tamoxifen-induced GABA_B_R downregulation and GCaMP3 expression in NG2^+^ OPCs, spinal cord window implantation surgery, and in vivo two-photon laser-scanning microscopy (2P-LSM). (**B**) Representative maximum-intensity projections of the GCaMP3 signal (ΔF/F0) for representative FOVs over the entire recording time (up to 5 min), and associated maps of selected identified regions-of-activity (ROAs) with heatmap representation of the GCaMP3 signal profile over time. Scale bar, 20 µm; 30 s. (**C**) Signal density and (**D**) ROA density expressed as the number of signals/ROAs detected over the entire recording time. (**E**) Per-ROA signal frequency. (**F**) ROA area. (**G**) Signal amplitude in correspondence of the signal maximum. (**H**) Signal duration expressed as full width at half maximum (FWHM). Data are represented as mean ± SEM and are derived from N = 3–4 mice (*n* = 12 FOVs). Data were analyzed using an unpaired *t* test. *, *p* < 0.05; ** *p* < 0.01 and **** *p* < 0.0001.

The accumulating evidence revealed that Ca^2+^ signaling is a key regulator of OPC proliferation and differentiation [39,40], as well as myelination [35,36,37,67]. In particular, it has been recently suggested that OPC Ca^2+^ activity is reduced along their differentiation to mature oligodendrocytes [40]. In virtue of these considerations, and given the putative role of GABA_B_Rs on the regulation of intracellular Ca^2+^ concentration and signaling [22], we used in vivo two-photon laser scanning microscopy (2P-LSM) to record the spontaneous OPC Ca^2+^ signaling of the dorsal spinal cord white matter tracts to evaluate if the differences in oligodendrocyte-lineage cells and the differential response to demyelination in control and cKD mice could be due to underlying differences in OPC Ca^2+^ (Figure 6A,B). OPC-GABA_B_R cKD mice displayed a drastic increase in the number of recorded Ca^2+^ oscillations with three times more signals than controls (ctrl, 17.78 ± 3.81 10^−2^ µm^−2^min^−1^; cKD, 57.80 ± 6.12 10^−2^ µm^−2^min^−1^; *p* < 0.001) (Figure 6C). The increased signal number resulted from an increased spatial signal density, as the regions-of-activity (ROAs) detected in the field-of-view showed a 1.5-fold increase in density (ctrl, 24.16 ± 2.74 10^−3^ µm^−2^min^−1^; cKD, 34.21 ± 3.76 10^−3^ µm^−2^min^−1^; *p* = 0.007) (Figure 6D), as well as from an increased temporal density, as the per-ROA signal frequency also displayed a 1.6-fold increase (ctrl, 3.86 ± 0.68 min^−1^; cKD, 6.29 ± 0.41 min^−1^; *p* = 0.002) (Figure 6E). On the other hand, the ROA area of the automatically detected Ca^2+^ signals was reduced by ~ 57 % in cKD mice (ctrl, 82.80 ± 14.92 µm^2^; cKD, 35.26 ± 6.37 µm^2^; *p* = 0.003) (Figure 6F), indicating that the increased Ca^2+^ activity in cKD mice is paralleled by the reduction in its spatial extension. Finally, we did not detect any difference in the signal amplitude between ctrl and cKD mice (Figure 6G) but a slight reduced signal duration in the cKD group (ctrl, 4.98 ± 1.00 s; cKD, 3.17 ± 0.06 s; *p* = 0.044) (Figure 6H). This suggests that the genetic downregulation of GABA_B_Rs in OPCs impacts the spatial and temporal distribution of OPC Ca^2+^ signaling, resulting in increased Ca^2+^ oscillations with a reduced spatial extension and duration.

### 3.6. OPC-GABA_B_R Downregulation Counters Cuprizone-Induced Alterations in OPC Ca^2+^ Signaling

**Figure 7:**
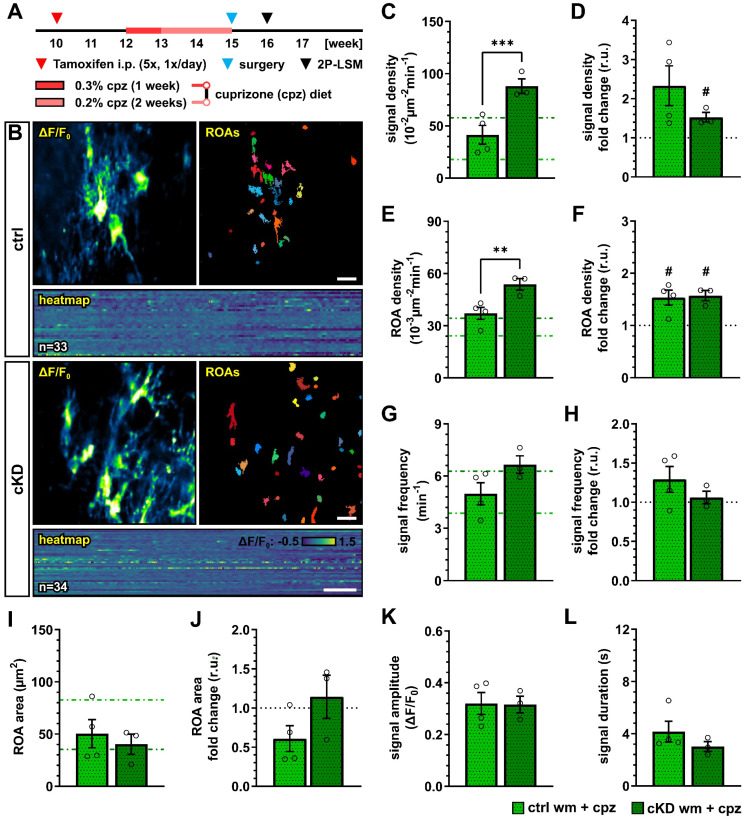
Cuprizone-induced alterations in OPC Ca^2+^ signaling are reduced after OPC-GABA_B_R downregulation. (**A**) Experimental design for tamoxifen-induced GABA_B_R downregulation and GCaMP3 expression in NG2^+^ OPCs, cuprizone treatment, spinal cord window implantation surgery, and in vivo two-photon laser-scanning microscopy (2P-LSM). (**B**) Representative maximum-intensity projections of the GCaMP3 signal (ΔF/F0) for representative FOVs over the entire recording time (up to 5 min), and associated maps of selected identified regions-of-activity (ROAs) with associated heatmap representation of the GCaMP3 signal profile over time. Scale bar, 20 µm; 30 s. (**C**) Signal density and (**D**) fold-change compared to untreated ctrl and cKD mice. (**E**) ROA density and (**F**) fold-change. (**G**) Per-ROA signal frequency and (**H**) fold-change. (**I**) ROA area and (**J**) fold-change. (**K**) Signal amplitude and (**L**) signal duration expressed as full width at half maximum (FWHM). Data are represented as mean ± SEM and are derived from N = 3–4 mice (*n* = 12 FOVs). In (**C**,**E**,**G**,**I**), data from untreated groups are overlapped as dotted lines (ctrl, light green; cKD, dark green). Data were analyzed using an unpaired *t* test. For each group in (**D**,**F**,**H**,**J**), data were tested using a one-sample *t* test to compare their mean with the hypothetical value μ = 1. In (**D**,**F**,**H**,**J**), the black dotted line corresponding to the non-treated ctrl and cKD (fold change = 1) is included as reference. # *p* < 0.05; ** *p* < 0.01 and *** *p* < 0.001.

We next investigated how the cuprizone treatment alters the OPC Ca^2+^ activity in control and OPC-GABA_B_R cKD mice. To this aim, we implanted a spinal cord window on 15-week-old mice immediately at the end of the cuprizone treatment and recorded with 2P-LSM the spontaneous Ca^2+^ activity from the white matter tracts one week later (Figure 7A,B). As we observed in the untreated groups, GABA_B_R cKD mice also displayed, under pathological conditions, an increased OPC Ca^2+^ signal density (ctrl, 41.41 ± 9.02 10^−2^ µm^−2^ min^−1^; cKD, 88.13 ± 7.03 10^−2^ µm^−2^ min^−1^; *p* < 0.001) (Figure 7C). Compared to the untreated-groups, the OPC Ca^2+^ signal density was increased by at least 1.5-fold, with a larger increase in control mice (ctrl, 2.33 ± 0.51, ns, *p* = 0.079; cKD, 1.53 ± 0.12, *p* = 0.049) (Figure 7D). Similarly, the control and GABA_B_R cKD mice were associated with an increased ROA density (ctrl, 37.08 ± 3.46 10^−3^ µm^−2^ min^−1^; cKD, 53.82 ± 3.34 10^−3^ µm^−2^ min^−1^; *p* = 0.006) (Figure 7E) with a similar 1.5-fold change increment compared to untreated conditions in both groups (Figure 7F). After cuprizone treatment, the signal frequency of the OPC Ca^2+^ activity was similar between control (4.99 ± 0.64 min^−1^) and GABA_B_R cKD mice (6.66 ± 0.50 min^−1^, *p* = 0.082) (Figure 7G), resulting from the specific increase in OPC Ca^2+^ activity in control mice compared to untreated conditions (Figure 7H). On the other hand, the ROA spatial extension also did not show any difference after cuprizone treatment between control and cKD mice (ctrl, 50.36 ± 13.59 µm^2^; cKD, 40.29 ± 9.70 µm^2^; *p* = 0.593) (Figure 7I) resulting from the specific decrease in the ROA area in control mice compared to untreated conditions (ctrl, 0.61 ± 0.16, ns, *p* = 0.097; cKD, 1.14 ± 0.28, *p* = 0.656) (Figure 7J). Finally, the signal amplitude or duration after cuprizone treatment was similar between control and cKD mice (Figure 7K,L), as well as compared to untreated mice. Taken together, our data suggest that the cuprizone treatment induces an increase in OPC Ca^2+^ activity and that the cKD of OPC-GABA_B_Rs partially counteracts the pathological shift in OPC Ca^2+^ signaling activity.

## 4. Discussion

In this work, we took advantage of the time- and spatial-controlled, cell-type specific GABA_B_R downregulation in NG2^+^ OPCs to selectively evaluate their contribution to the OPC proliferation, differentiation, and maturation in the murine spinal cord. Evidence on the specific role of GABA_B_R signaling in the context of oligodendrocyte-lineage cell physiology is currently limited to in vitro studies [26,30], and to the brain [28] or developmental stages [32]. Therefore, the transposition of previous results obtained in the field to the spinal cord is challenging, especially by virtue of the high heterogeneity of OPCs across age and CNS location, which has been extensively reported and reviewed [68,69,70,71]. Studies addressing the role of GABA_B_Rs in the spinal cord have been using the GABA_B_R agonist baclofen [31]. Given the use of baclofen in the therapy of spasticity associated with MS, these results are of key importance. Nevertheless, they still lack the cell specificity required to identify the specific contribution of OPC-GABA_B_Rs. On the other hand, it is true that both GABA_B_1 and GABA_B_2 subunits are downregulated during OPC differentiation to mature oligodendrocytes (at least in vitro) [30]. Our approach, however, selectively targets OPCs with high efficiency, without significantly altering the receptome and physiology of mature oligodendrocytes with the exception of oligodendrocyte newly generated from recombined OPCs (which, at the time point of analysis and in the spinal cord, never contributed to more than 10% of existing mature oligodendrocytes) (Supplementary Figure S1E–G).

Our results showed that, in the adult spinal cord, the conditional OPC specific GABA_B_R knock-down (cKD) does not affect the total number of OPCs and mature oligodendrocytes (in neither the gray nor the white matter). Nevertheless, specifically in the white matter, OPC differentiation seems to be reduced, as indicated by the reduction in the ratio between the recombined mature oligodendrocytes and OPCs (Figure 1). It has been recently shown that OPC and OPC-GABA_B_R signaling are key players in cortical myelination during development but not in the adult brain [28]. In line with this, we also did not observe any reduction in spinal myelination in our adult OPC-GABA_B_R cKD mice, neither in terms of MBP coverage nor in terms of myelin thickness (Figure 2). To address the contribution of OPC-GABA_B_Rs in the cellular response to pathological demyelinating insults of the spinal cord, we took advantage of two different models for toxic demyelination, namely, the cuprizone and the lysolecithin models. The cuprizone model mimics several aspects of human MS and is associated with a less extensive primary immune response compared to other models including the lysolecithin model [58,59], but its use in the spinal cord has been so far limited due to a reduced response compared to forebrain white matter [60,61,62]. Indeed, in line with these observations, we found no specific increase in astrogliosis or microgliosis upon cuprizone treatment (Supplementary Figure S3), as we did not detect any enhanced OPC proliferation in the spinal cord. Nevertheless, the cuprizone treatment effectively reduced the cell density of the mature oligodendrocyte in the white matter spinal cord (Figure 3). This result mirrors the higher susceptibility of mature oligodendrocytes (by virtue of their high energy demand) to the copper-chelating action of cuprizone [13,72]. On the other hand, OPC-GABA_B_R cKD mice showed no reduction in the number of mature oligodendrocytes, in line with the protective role of the genetic downregulation of OPC GABA_B_Rs (Figure 3). Nevertheless, it is not clear from these results if the protective effect of the downregulation of OPC GABA_B_Rs is to be ascribed to an enhanced OPC differentiation or to a higher resilience of mature oligodendrocytes. To note, OPC differentiation in the cKD mice is reduced under physiological conditions, suggesting that OPC GABA_B_R signaling may be distinctly involved in OPC differentiation in physiological and pathological conditions.

From a clinical point of view, more important than the enhanced oligodendrocyte survival is the subsequent partial rescue of the cuprizone-induced demyelination. OPC-GABA_B_R cKD mice showed no reduction in total MBP and no reduction in the MBP coverage, as was the case for cuprizone-treated control mice. On the other hand, the analysis of the MBP^+^ area surrounding single white matter axons revealed that both control and cKD mice experienced a reduction in MBP area but with a reduced extent in OPC-GABA_B_R cKD mice (Figure 4). These results suggest that cuprizone induces a thinning of the myelin rings in the white matter of the spinal cord of both groups, with OPC-GABA_B_R cKD mice showing a higher resistance to cuprizone. To note, the confocal myelin analysis provided in this work would greatly benefit from a microstructural analysis of the myelin rings, which could be assessed by means of an electron microscopic analysis of the g-ratio as well as an evaluation of structural alterations induced by cuprizone.

Similar results were obtained from the MBP evaluation in the dorsal white matter upon local lysolecithin treatment (Figure 5). Contrary to the cuprizone model, the loss of MBP was associated with a strong astro- and microglial reaction in control and cKD mice (Supplementary Figure S4), suggesting that the lysolecithin model may be preferred to the cuprizone model for studies on demyelination in the spinal cord. Moreover, this may be due to the different modes of action of cuprizone and lysolecithin, since lysolecithin, but not cuprizone, acts as a direct activator for immune cells [64,65,66]. Nevertheless, beyond their differences, in both models, the loss of MBP was reduced in OPC GABA_B_R cKD mice, suggesting that the protective role of OPC GABA_B_R cKD on demyelination may be independent of its specific trigger. To note, in the lysolecithin model, OPCs are recruited to the lesion site, at the earliest, five days after lesion induction, but are reported to differentiate only from day 10 [73,74,75,76], suggesting that the observed results may not underly a differential differentiation capacity of cKD OPCs but a higher resilience to demyelination of the oligodendrocyte lineage.

Although the results obtained using the lysolecithin model seem in contradiction to what has been shown using the GABA_B_R agonist baclofen using the same model in the spinal cord [31], it is worth mentioning that, in our study, we induced the cell-type-specific GABA_B_R downregulation before the lesion and focused on the demyelination phase of the model, whereas Serrano-Regal et al. activated GABA_B_R globally after the lesion and focused on remyelination. Therefore, our combined results clearly indicate a complex contribution of GABA_B_R signaling in the context of de- and remyelination in the spinal cord.

This is also highlighted by the complex changes in OPC Ca^2+^ signaling under physiological and pathophysiological conditions. The downregulation of OPC-GABA_B_Rs under physiological conditions resulted in the alteration of OPC Ca^2+^ oscillations, leading to a higher spatial incidence and frequency of Ca^2+^ signals but with a reduced spatial (area) and temporal extension (duration) (Figure 6). Glial Ca^2+^ signaling is, as it is in other regions of the CNS, finely regulated and cell-specific in the mouse spinal cord [50], but we are far from understanding its determinants as well as its role in cell physiology. In the functional spinal cord, OPC Ca^2+^ signals are known to be evoked by neurons and astroglia [24], and disturbances in OPC Ca^2+^ (with excessively frequent Ca^2+^ oscillations) have been linked to impaired proliferation during development [77,78]. Compared to physiological conditions in cKD mice, OPCs showed a higher resilience to the pathological alteration of cellular Ca^2+^ signaling induced by the cuprizone treatment. In fact, control OPCs displayed increased Ca^2+^ signaling and reduced Ca^2+^ spatial extension (like the cKD under physiological conditions), which was partially counteracted in cKD mice (Figure 7). It has been shown that a sustained Ca^2+^ increase resulting from the activation of P2 × 7 purinergic receptors in oligodendrocytes leads to oligodendrocyte death and demyelination in vivo [67] and that low-frequency Ca^2+^ transients with a longer duration in oligodendrocytes are associated with myelin sheath shortening [36,37], which are in line with our results obtained in OPCs and support the use of a common Ca^2+^-coded language for the cells of the oligodendrocyte lineage. To date, little is known about the specific contribution of OPC Ca^2+^ in the context of demyelination. Nevertheless, it has been shown in the spinal cord of zebrafish [39] that the higher Ca^2+^ activity in OPC is associated with an enhanced proliferation and reduced differentiation, whereas a lower Ca^2+^ activity is associated with enhanced differentiation. Moreover, it has recently been shown that, in the somatosensory cortex of awake-behaving mice, the OPC Ca^2+^ activity decreases as they differentiate into oligodendrocytes [40]. In line with this, we found that OPC Ca^2+^ activity increases upon cuprizone treatment and is associated with a reduced OPC differentiation and that the downregulation of OPC GABA_B_Rs counteracts the cuprizone-induced pathological alteration of OPC Ca^2+^ activity and is associated with an enhanced OPC differentiation upon cuprizone treatment.

Although our results cannot clarify the contribution of OPC Ca^2+^ to the cell fate and myelination, we could show that the resilience to pathological alterations of Ca^2+^ in cKD OPCs was associated with a higher endurance to demyelinating insults. It is to be noted that the differences between control and cKD OPCs even in physiological conditions may open up different scenarios, including a pre-existing OPC alteration which masks cuprizone-induced changes, or, conversely, a pre-existing priming of cKD OPCs which could make them more prone to readily reacting to demyelination insults. Moreover, OPC differentiation and oligodendrocyte maturation are affected by other intracellular pathways than the Ca^2+^ signaling cascade (including but not limited to the MAP kinase and the mTOR pathways) [79]. The contribution of parallel intracellular pathways influencing the cellular response to the genetic downregulation of OPC GABA_B_Rs in the pathophysiology of the spinal cord would shine a light on the eventual compensatory mechanisms acting on the link between Ca^2+^ signaling and the physiology of the oligodendrocyte cell lineage.

## 5. Conclusions

Summarized, our study evaluated the role of OPC-GABA_B_Rs signaling in oligodendrocyte-lineage cells in the mouse spinal cord. Despite the accumulating evidence on the involvement of OPC responses to GABAergic signaling in the CNS homeostasis and the response to demyelinating insults, the specific contribution of OPC-GABA_B_Rs in vivo remains elusive. Through the targeted genetic downregulation of GABA_B_Rs from OPCs, we showed that their absence does not alter the density of OPC and mature oligodendrocytes under physiological conditions in the adult spinal cord. However, the conditional knock-down of OPC-GABA_B_Rs leads to distinctive alterations in OPC Ca^2+^ signaling, emphasizing the intricate regulatory role of GABA_B_Rs in the physiology of oligodendrocyte-lineage cells. Furthermore, our examination of the cuprizone and lysolecithin models of toxic demyelination uncovered a protective effect associated with the downregulation of OPC-GABA_B_Rs. OPC-GABA_B_R cKD mice exhibited an enhanced resilience to cuprizone-induced alterations in OPC Ca^2+^ signaling and, more importantly, a partial rescue of mature oligodendrocyte loss and demyelination. These findings not only shed new light on the involvement of GABA_B_Rs in OPC physiology but also hint at potential therapeutic implications in the context of demyelinating disorders. Overall, our study provides a crucial foundation for the further unraveling of the intricate dynamics of GABA_B_R signaling in spinal cord oligodendrocyte-lineage cells and their response to demyelination.

## Data Availability

Minimal data is contained within the Supplementary Material. Further inquiries on the raw data can be directed to the corresponding author.

## Supplementary Materials

The following supporting information can be downloaded at: https://www.mdpi.com/article/10.3390/cells13232014/s1, Figure S1: OPC-specific conditional knock-down of GABABRs. Figure S2: Early tamoxifen administration results in increased number of recombined mature oligodendrocytes. Figure S3: GFP^−^ OPC and mature oligodendrocyte cell density is unaffected in cKD mice. Figure S4: Effect of OPC GABABR down regulation on cell density of non-recombined oligodendrocyte-lineage cells upon cuprizone treatment. Figure S5: No glial response in terms of astroglial GFAP expression or microglia number in the spinal cord after cuprizone treatment. Figure S6: Acute LPC incubation induces strong glial response in the white matter of the spinal cord of control and cKD mice. Table S1: Primers used for qRT-PCR. Oligonucleotides are listed in the 5′-3′ direction.
