# Supplementary material for: Genetic Downregulation of GABAB Receptors from Oligodendrocyte Precursor Cells Protects Against Demyelination in the Mouse Spinal Cord
Source: Cells. 2024 Dec 5;13(23):2014. doi: 10.3390/cells13232014 (PMC11640606; doi:10.3390/cells13232014)
Supplement: Supplementary file 1 [file cells-13-02014-s001.zip › Supplementary Materials.pdf]

Article

Genetic down regulation of $GABA_B$ receptors from oligodendrocyte precursor cells protects against demyelination in the mouse spinal cord

Davide Gobbo, Phillip Rieder, Li-Pao Fang, Emeline Buttigieg, Moritz Schablowski, Elisa Damo, Nathalie Bosche, Eleonora Dallorto, Pascal May, Xianshu Bai, Frank Kirchhoff and Anja Scheller

Supplementary Materials

The following supporting information can be downloaded at: www.mdpi.com/xxx/s1, Supplementary Figures S1-S6; Supplementary Table S1.

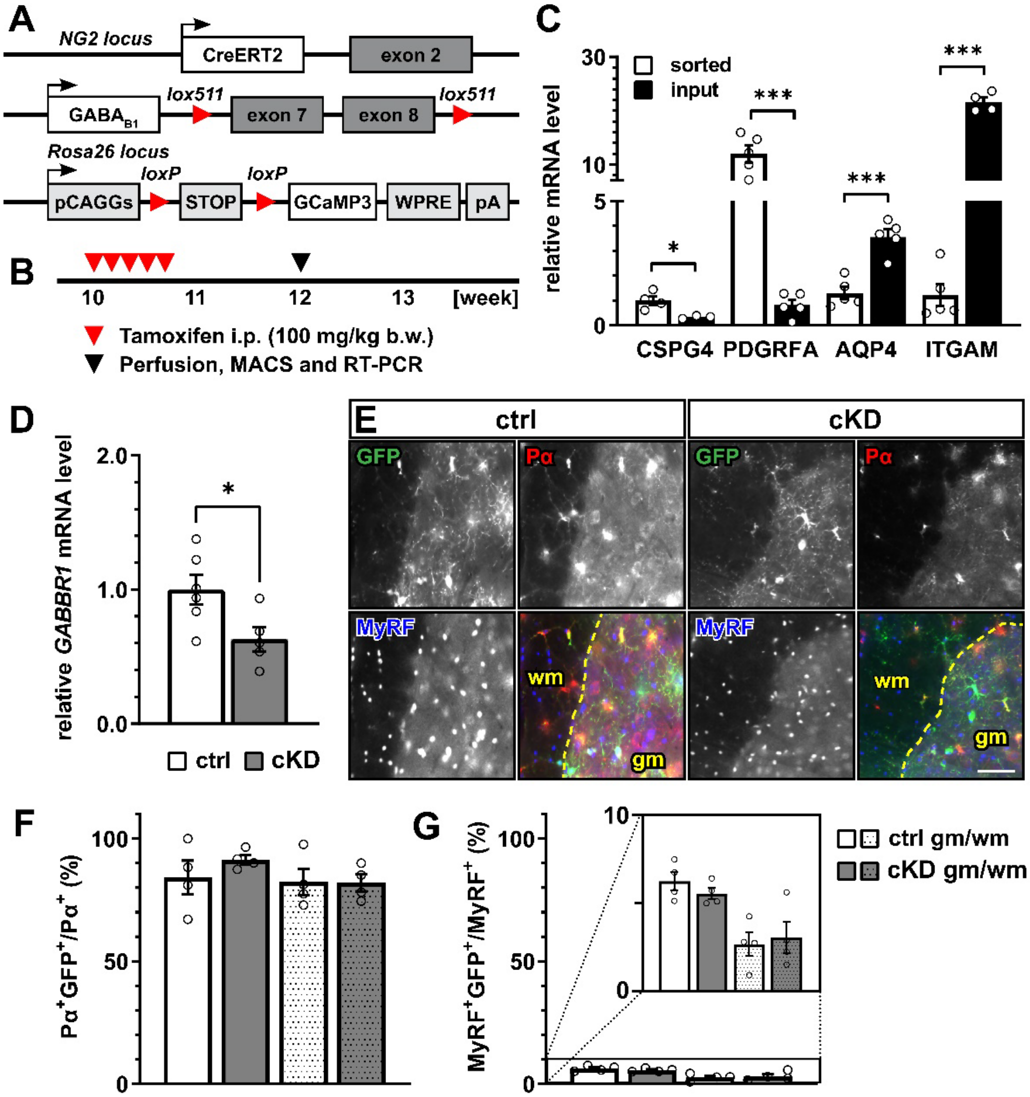

Supplementary Figure S1. OPC-specific conditional knock-down of GABA$_B$Rs. (A) Schematic representation of genetic knock-in manipulation required for the tamoxifen-induced GABA$_B$R down regulation and GCaMP3 (GFP) expression in NG2$^+$ OPCs. (B) Experimental design for tamoxifen treatment, perfusion, Magnetic Cell Separation Sorting (MACS) and real-time PCR (RT-PCR). (C) Cell enrichment analysis of sorted and unsorted (input) cells from spinal homogenate using RT-PCR for OPC- (chondroitin sulfate proteoglycan 4, CSPG4; PDGFRA), astroglial- (aquaporin-4, AQP4) and immune cell- (integrin α M, ITGAM) specific markers. (D) RT-PCR of GABBR1 mRNA in spinal homogenates from control (ctrl) and OPC-GABA$_B$R conditional knock-out (cKD) mice. (E) Immunohistochemistry (IHC) of spinal cord tissue stained for GFP (green), PDGFRα (Pα, red) and MyRF (blue). Scale bar, 20 µm. (F) Percentage of recombined OPCs (Pα^+GFP$^+$) on total number of OPCs (Pα^+). (G) Percentage of recombined mature oligodendrocytes (MyRF$^+$GFP$^+$) on total number of mature oligodendrocytes (MyRF$^+$). Data are represented as mean ± SEM and derive from N=3-6 mice for the MACS experiments or N=4 (n=12 FOVs) for IHC. Data were analyzed using a two-way ANOVA with multiple comparisons test (C, F-G) or an unpaired t test (D).

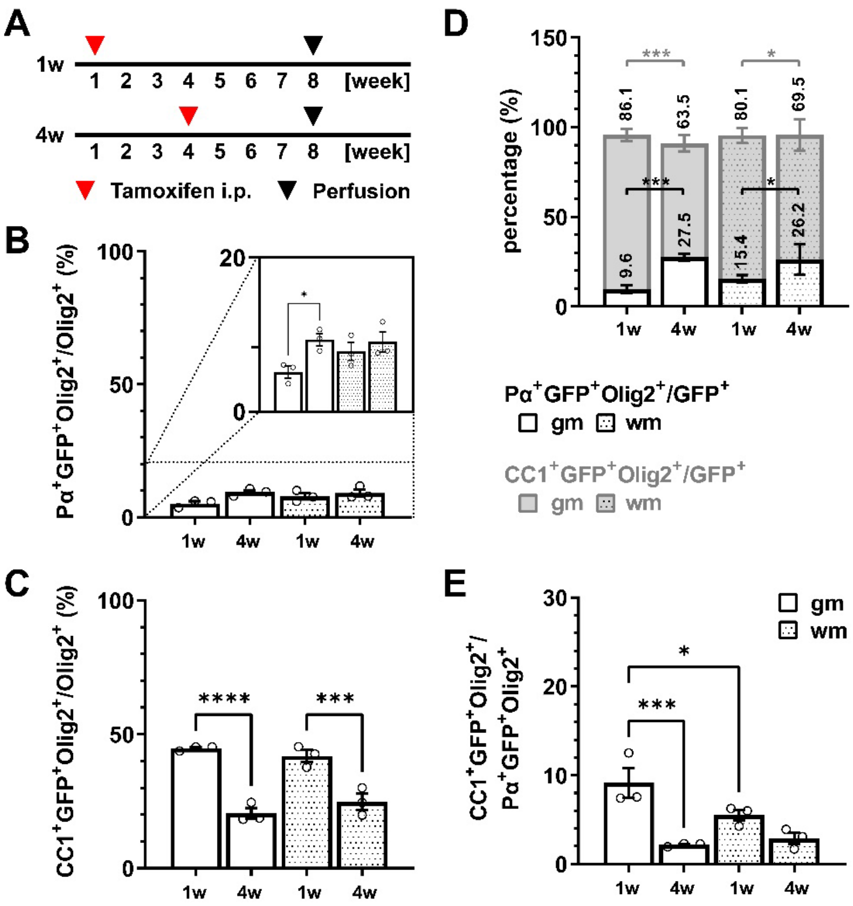

Supplementary Figure S2. Early tamoxifen administration results in increased number of recombined mature oligodendrocytes. (A) Experimental design for tamoxifen-induced GCaMP3 (GFP) expression in NG2+ OPCs either at postnatal week one (1w, 2x, 1x/day, i.p.) or postnatal week four (4w, 5x, 1x/day, i.p.). Perfusion and IHC analysis were performed at postnatal week 8. (B) Percentage of recombined PDGFR*α* (P*α*)+GFP+Olig2+ OPCs on the total number of Olig2+ oligodendrocyte-lineage cells. (C) Percentage of recombined CC1+GFP+Olig2+ mature oligodendrocytes on the total number of Olig2+ oligodendrocyte-lineage cells. (D) Proportion of recombined OPCs and mature oligodendrocytes in the total pool of recombined cells (GFP+). (E) Ratio between recombined oligodendrocytes and recombined OPCs. Data are represented as mean ± SEM and derive from N=3 mice (n=12 FOVs). Data were analyzed using a Two-way ANOVA with multiple comparisons test.

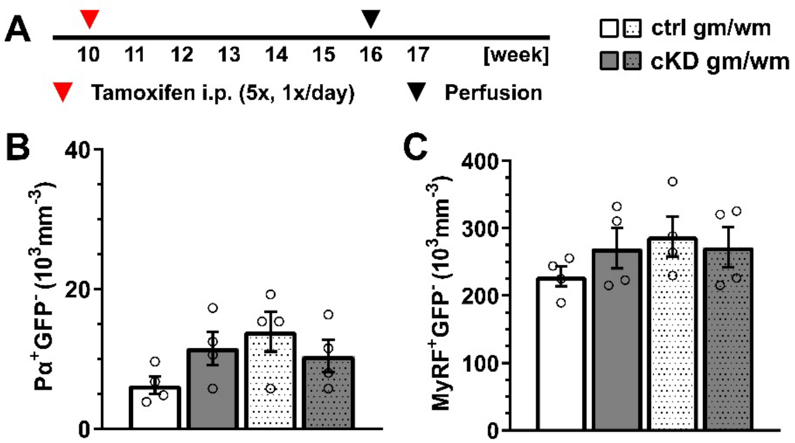

Supplementary Figure S3. GFP- OPC and mature oligodendrocyte cell density is unaffected in cKD mice. (A) Experimental design for tamoxifen-induced GABABR down regulation and GCaMP3 (GFP) expression in NG2+ OPCs, perfusion and immunohistochemical analysis (IHC). Non-recombined OPC (Pα^+GFP$^-$, C) and mature oligodendrocyte (MyRF$^+$GFP$^-$, D) cell density in the gray (gm) and white matter (wm) of the spinal cord. Data are represented as mean ± SEM and derive from N=3-4 mice (n=12 FOVs). Data were analyzed using a Two-way ANOVA with multiple comparisons test.

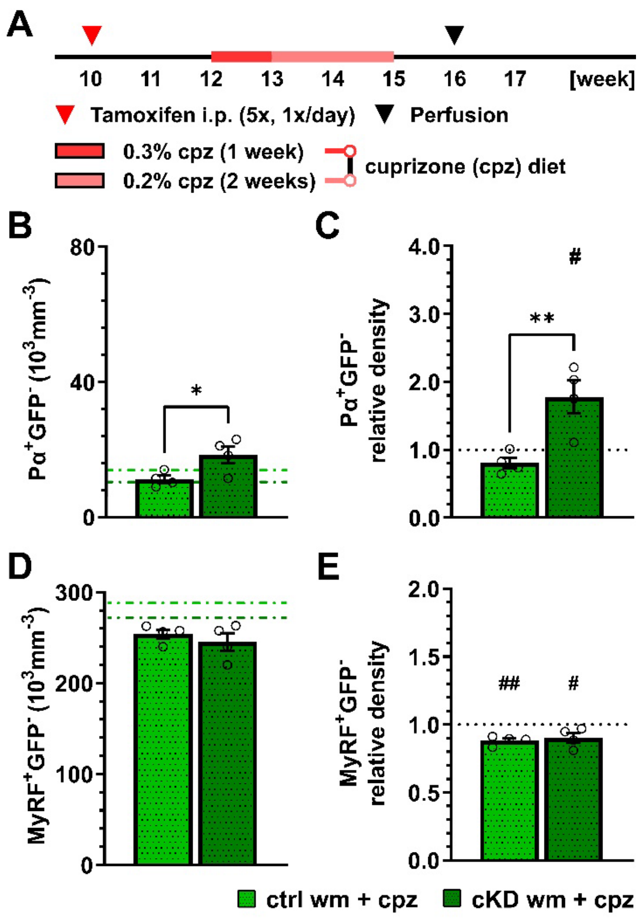

Supplementary Figure S4. Effect of OPC GABABR down regulation on cell density of non-recombined oligodendrocyte-lineage cells upon cuprizone treatment. (A) Experimental design for tamoxifen-induced GABABR down regulation and GCaMP3 (GFP) expression in $NG2^+$ OPCs, cuprizone (cpz) treatment, perfusion and IHC analysis of ctrl and cKD mice. Non-recombined OPC ($P\alpha^+GFP^-$, B) cell density and fold-change (C) compared to untreated ctrl and cKD mice. Non-recombined mature oligodendrocyte ($MyRF^+GFP^-$, D) cell density and fold-change (E). Data are represented as mean ± SEM and derive from N=3-4 mice (n=12 FOVs). In B and D, data from untreated groups are overlapped as dotted lines (ctrl, light green; cKD, dark green). Data were analyzed using an unpaired t test. For each group in C and E, data were tested using a one sample t test to compare their mean with the hypothetical value μ=1.

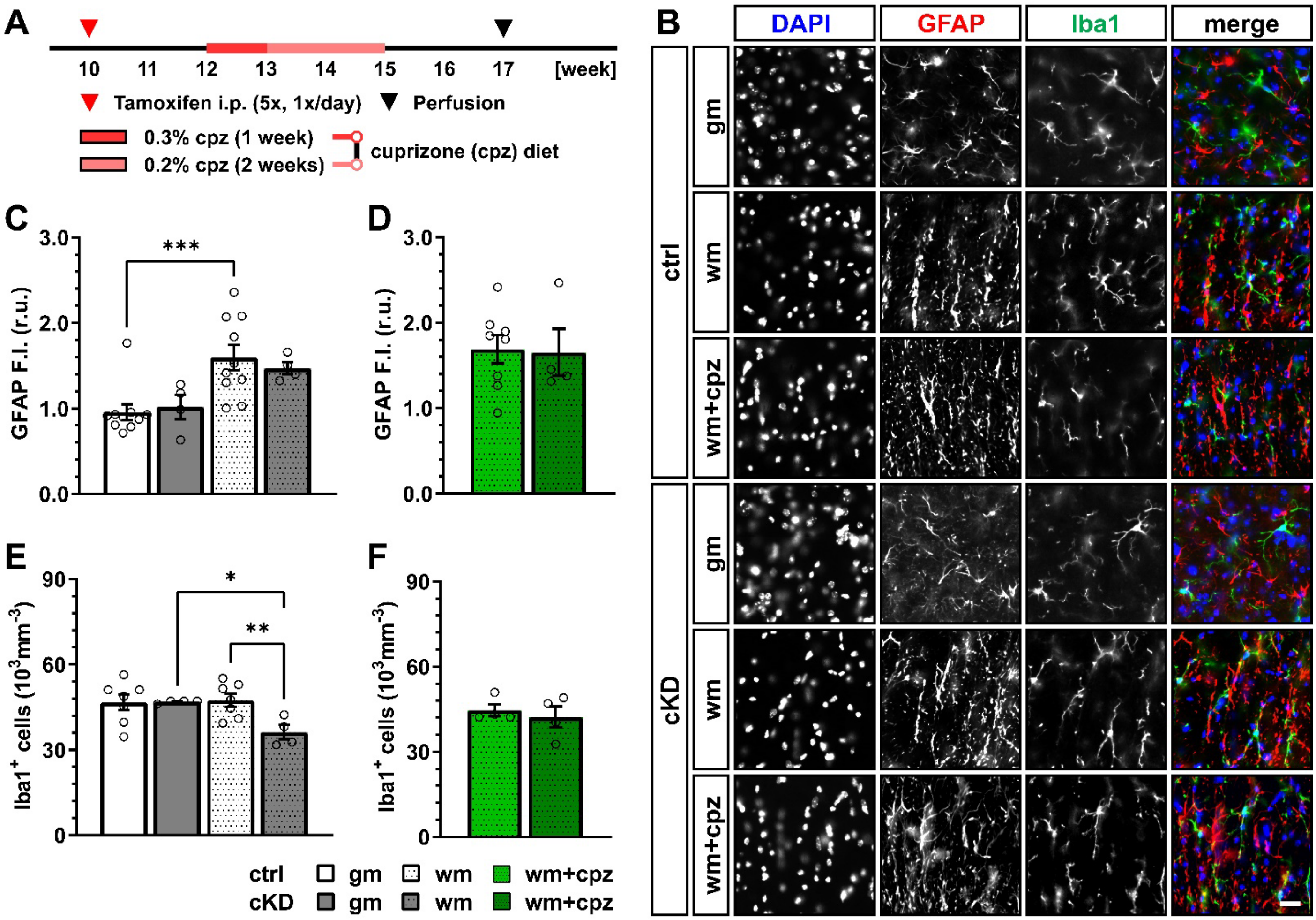

Supplementary Figure S5. No glial response in terms of astroglial GFAP expression or microglia number in the spinal cord after cuprizone treatment. (A) Experimental design for tamoxifen-induced GABABR down regulation and GCaMP3 expression in NG2+ OPCs, cuprizone (cpz) treatment, perfusion and IHC analysis. (B) IHC of spinal cord tissue from untreated and cuprizone-treated ctrl and cKD mice (gray matter, gm; white matter, wm) stained for DAPI (blue), Glial Fibrillary Acidic Protein (GFAP, red) and Iba1 (green). Scale bar, 20 µm. Mean fluorescence intensity (F.I.) of GFAP signal in untreated (C) and cpz-treated (D) mice. Iba1+ cell density in untreated (E) and cuprizone-treated (F) mice. Data are represented as mean ± SEM and derive from N=3-8 mice (n=12-32 FOVs). Data were analyzed using a two-way ANOVA with multiple comparisons test (C, E) or an unpaired t test (D, F).

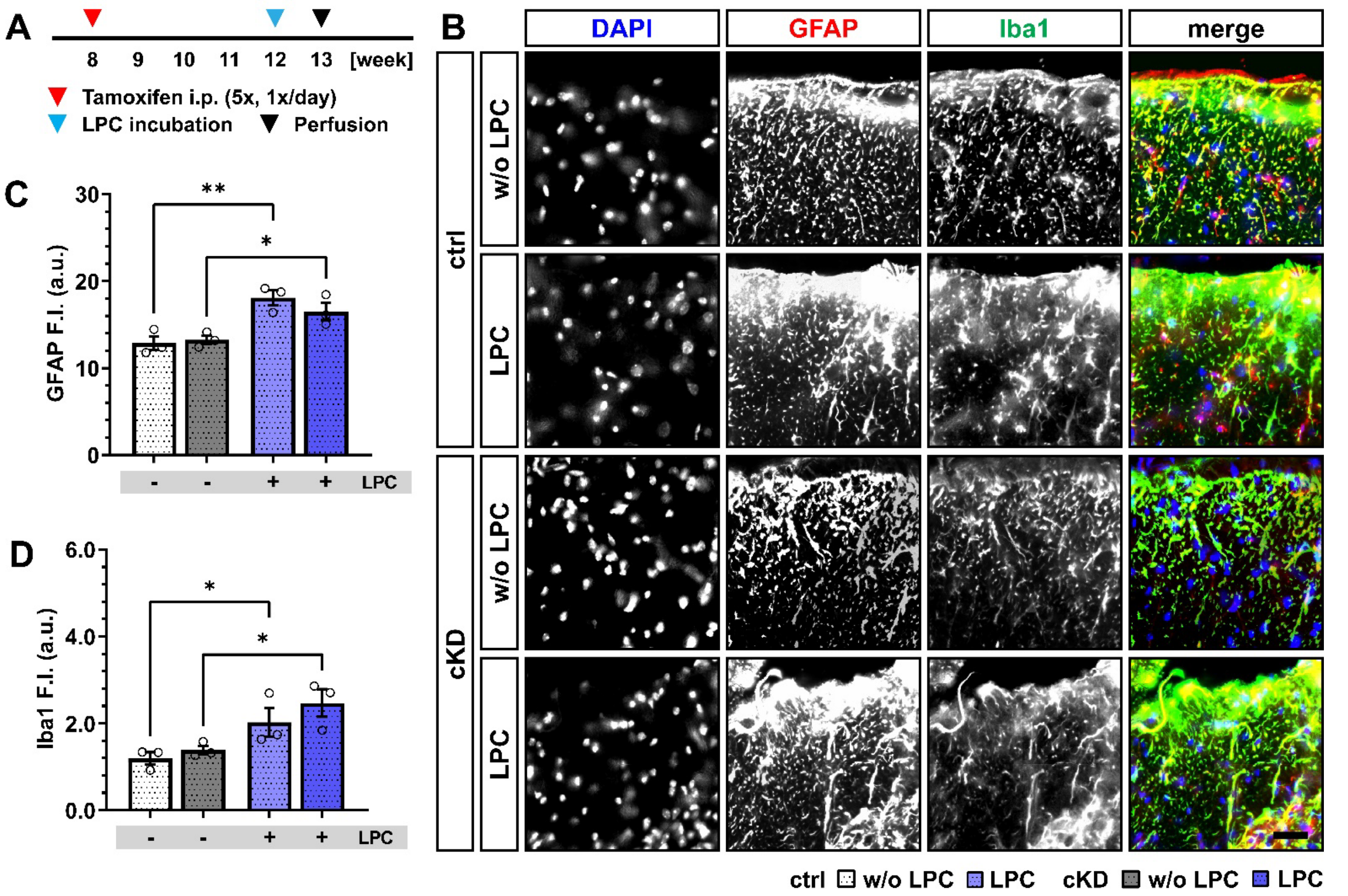

Supplementary Figure S6. Acute LPC incubation induces strong glial response in the white matter of the spinal cord of control and cKD mice. (A) Experimental design for tamoxifen-induced GABA$_B$R down regulation and GCaMP3 (GFP) expression in NG2$^+$ OPCs, lysolecithin (LPC) incubation, perfusion and IHC. (B) IHC of the dorsal white matter of spinal cord tissue from untreated and LPC-treated ctrl and cKD mice stained for DAPI (blue), GFAP (red) and Iba1 (green). Scale bar, 20 µm. (C) Mean fluorescence intensity (F.I.) of GFAP signal in LPC-treated mice. (D) Mean fluorescence intensity (F.I.) of Iba1 signal in LPC-treated mice. Data are represented as mean ± SEM and derive from N=3 mice (n=12 FOVs). Data were analyzed using a two-way ANOVA with multiple comparisons test.

Supplementary Table S1. Primers used for qRT-PCR. Oligonucleotides are listed in the 5′-3′ direction.

Gene	Forward	Reverse
AQP4	5'-CTTTCTGGAAGGCAGTCTCAG-3'	5'-CCACACCGAGCAAAACAAAGAT-3'
CSPG4	5'-GGGCTGTGCTGTCTGTTGA-3'	5'-TGATTCCCTTCAGGTAAGGCA-3'
GABBR1	5'-CGAAGCATTTCCAACATGAC-3'	5'-CAAGGCCCAGATAGCATCATA-3'
ITGAM	5'-ATGGACGCTGATGGCAATACC-3'	5'-TCCCCATTCACGTCTCCCA-3'
PDGFRA	5'-TCCTTCTACCACCTCAGCGAG-3'	5'-CCGGATGGTCACTCTTTAGGAAG-3'
